# The International Homœopathic Congress of Paris, August 21st, 22d, and 23d, 1889

**Published:** 1890-04

**Authors:** 


### THE INTERNATIONAL HOMŒOPATHIC CONGRESS OF PARIS, AUGUST 21st, 22d, and 23d, 1889.

### SUMMARY OF ITS PROCEEDINGS.

Translated from the Spanish of M. Cahis, by E. A. P.

The matters under discussion at the double sessions of the three days were as follow:

*Materia Medica and Therapeutics.*—1. Contrary effects of medicines upon the sick and upon the well, and their relation to the doses, Dr. Piedvache, of Paris. 2. Relations between the germ theory and the homœopathic therapeutics, Dr. P. Jousset, Paris. 3. Homœopathic therapeutics and its relations to other branches of therapeutics, Dr. Pinilla, Madrid. 4. The method of studying pure materia medica, Dr. Gailliard, Brussels. 5. Use of electricity in homœopathic medicine, Dr. Conan, Paris.

*Application of Materia Medica to Therapeutics.*—1. Homœopathic treatment of Bright's disease, Dr. Hansen, Copenhagen. 2. Treatment of locomotor ataxia, and of the state of pseudotabes, Dr. von Villers, Dresden. 3. Of the possible cure of the cancer diathesis, Dr. Criquelion, Mons. 4. Treatment of cancerous tumors by homœopathic medicines, Dr. Gutteridge, London. 5. Of Hydrastis canadensis in mammary cancers and infarcted glands of this organ, Dr. Imbert de la Touche, Lyons. 6. Therapeutic employment of some new remedies under the law of the simillimum, Dr. Ozanam, Paris. 7. Of massive doses of Caffeine in the treatment of insomnia and neuralgia, Dr. Jousset, Jr., Paris. 8. Iritis and irido-choroiditis occurring in uterine affections, Dr. Parenteau, Paris. 9. Cure of a case of diphtheria with Cyanide of Mercury, by Dr. Serrand, Paris. 10. Croup and diphtheria, by Dr. Oxford, Lexington, U. S. A. 11. Seven observations on cures of senile trembling and paralysis agitans, by Dr. Imbert de la Touche, Lyons. 12. Pulmonary consumption in Algeria, statistics and treatment, by Dr. Feuillet, Algeria. 13. Diseases of women, diagnostics and cures, by Dr. Blake, London. 14. Homœopathic therapeutics of pregnancy, by Miss H. Keating, M. D., New York. 15. Homœopathic therapeutics applied to the especial diseases of women, by Miss Isabel Rankine, New York. 16. Some points on surgery, by Dr. Watson, London.

*Legislation.—Colleges and Hospitals.*—1. Mono-pharmacy, Dr. Gailliard, Brussels. 2. Propagation of and instruction in Homœopathy and its hospitals in Spain, Drs. Pellicer and Garia Lopez, Madrid. 3. Proceedings relating to secret medicines, by Rappaz, Montevideo. 4. Nomination of a commission of pharmacology, by Dr. Ecalle, Paris. 5. Homœopathy in the United States; rules for its practice in the State of Minnesota, by Dr. Ferrand, Paris. 6. Best methods of propagating Homœopathy, by Dr. Roth, London. 7. Homœopathic education of women in New York, Dr. Montague Lozier, New York. 8. Homœopathy in Cook County Hospital, by Dr. Gatchell, Chicago.

Owing to the large number of memorials and the limited time in which to consider them, together with the absence of several authors, it was agreed to confine the discussion to a limited number of subjects, as follows:

*Afternoon Session, August 21st.*—The President, Dr. P. Jousset, in the chair. The session opened at four o'clock. The President expressed his gratitude for the honor shown him, accepting it as a recompense for a life-long service that has been dedicated on all occasions to the defense of true therapeutics.

Much is hoped for the propagation of Homœopathy through this Congress, and it is believed that the themes under discussion will be most instructive.

The Congress will demonstrate that, faithful to the character impressed upon it by its founder, Homœopathy will energetically repel all pseudo-scientific systems that have hidden under its name practices of secret and mysterious therapeutics.

Polite and careful discussions only are desired.

On motion, Drs. Beck and Drysdale were elected Honorary Presidents.

Dr. Mark Jousset, General Secretary, read letters of excuse from absent members, and Dr. de Brasol's credentials as representative of the Homœopathic Society of St. Petersburg, and those of Drs. Helmuth, Wright, and McClelland representing the American Institute of Homœopathy.

It was announced that an International Homœopathic Congress would reassemble in the United States, and that Dr. Hughes, Perpetual Secretary of the five-years' Congress, would receive with much pleasure the assistance of those who desire to attend.

THE QUESTION OF THE DOSE.

The President invites the members of the Congress to take active part in the discussions on the works of materia medica and general therapeutics, adding that as Dr. Piedvache had not been able to finish his memorial on the contrary effects of medicine in sick and well patients, and of the same in their relations to the doses, he begged to be allowed to withdraw his conclusions, but it being such an important subject he invited its discussion.

Dr. Cigliano, of Naples, thinks that the absorption by the system of the remedy is subject to various laws; that when more attenuated its absorption is more rapid. This is accomplished in two ways: by the most important veins and by the lymphatics. The elimination is in two ways: rapidly by the urine, and more slowly by way of the veins and lymphatics. It is necessary to know these diverse operations, and when the moment is arrived to suspend the administration of the medicine, which is as soon as the absorbtion is completed. We thus avoid its accumulation in these channels of elimination where otherwise it would become dangerous. The effects of the medicines are not proportioned to the doses taken, but to the *amount absorbed.* Thus, a strong dose given once could not produce more than the effect of a small dose if only a minimum amount is absorbed, and, on the contrary, the weak doses repeated could produce the effects of a strong dose by internal accumulation.

Dr. Villers, of Dresden, remarked that if the administration of the medicine is limited to one dose, and waiting before renewing it if it be necessary to do so, then the accumulating symptoms that supervene should not be noted, but the critical symptoms from which we select the remedy.

The progress of pathology demonstrates that no disease exists without the involvement of the nerves. Why then cannot nervous diseases be cured with small doses? Why not infer from this that the small doses can cure *all* diseases and that we must return to the precepts of Hahnemann of prescribing the doses as small as possible and at long intervals?

Dr. P. Jousset says that on the question of doses the homœopaths are variously divided; some exclusively employ the high dilutions, others the strong, others both the one and the other, according to their cases. I formerly proposed a law to fix the doses that should be used according to the symptoms combated and according to the remedy, but it was not satisfactory, and I wish that some of the members would arrive at a decision that will assist the solution of the problem.

Dr. Liberali, of Rome, believes that it is not possible to give an absolute rule; and laments that there are brethren that daily employ massive doses, while others use only high dilutions, so high that they almost pass the margin of Hahnemann. The doses should be determined and subordinated to the kind of disease, the age and sex of the patient. Rome has intermittent fevers and serious pneumonias that necessitate at times dilutions relatively low.

Dr. Gailliard has cured, in Belgium, patients with marsh fever that resisted strong doses of sulphate of Quinine, by means of infinitesimal doses.

Dr. Cigliano believes that the important point is the individualizing of the remedy. When the remedy is well selected small doses only are needed; without them there is danger of aggravation. One day I prescribed for an English lady one drop of Lachesis $^{30}$. The patient took ten drops, at once causing such aggravation that she believed herself poisoned.

Dr. Gailliard remembers a law proposed in 1878 by Dr. Jousset that he thinks excellent. *It is necessary to choose that medicine the double action of which is similar to the pathological case that is to be combated; that is to say, we should use the infinitesimal doses to combat those symptoms which are analogous to the effects produced in a healthy person by infinitesimal doses, and use the ponderous doses against those symptoms which are analogous to those that are observed in the well person by strong and poisonous doses.* He proposes the study of this proposition for the next Congress. This motion was approved.

Dr. Léon Simón finds this question of doses most complicated. When he is in the presence of a patient the first thing is the selection of the remedy, next the choice of the dose, which, of course, differs undoubtedly with the case treated whether it be acute or chronic. With the former it would be promptitude, not contenting one's self with one dose a month. The variety of susceptibility in different individuals is worthy to be taken into consideration.

It is difficult to find a formula applicable to all cases, and it can be said with Hahnemann that in Homœopathy there is the absolute and variable. The absolute is the law of the similli-mum ; the variable is the choice of the dilution and the repetition.

Dr. Gallavardin, of Lyons, gives habitually a single dose, and allows it to work. The low dilutions have an action of short duration ; the high produce at times aggravation. Ordinarily begin with a medium dilution, and later go up to the 200th or higher.

Dr. Léon Simón has not used the very high dilutions, as very little is known of the way Jenichen and Korsakoff prepared them.

Dr. Vincent Léon Simón refers to experiments undertaken forty years ago in the Vienna hospitals. During two years the sixth dilution was given, two years later the fifteenth, and at last the thirtieth was given ; while in other hospitals they only used the first and second. The results were analogous, perhaps more flattering for the last hospital. The discussion here ended.

THE GERM THEORY AND ITS RELATION TO HOMŒOPATHY.

Dr. P. Jousset then read his paper on the *Relation of the doctrine of microbes to the homœopathic therapeutics.*

According to the microbe doctrine, all diseases are caused by a pathogenetic microbe. Disease then arises from external causes. The immediate consequence of this etiology is the application of antiseptic therapeutics, whose end is to destroy the microbes that cause the disease. These therapeutics are the result of the application of the axiom, *contraria contrariis curantur*, and its adoption the discarding of the homœopathic therapeutics as worthless.

The microbe theory is false as an etiological doctrine, because the microbe does not operate without the presence of a defined predisposition, and because many diseases that can be inoculated may be produced spontaneously by the work of a live organism.

Antiseptics are heroic medicines in traumatism but ineffectual for therapeutic cures. In medicine the part they play should be limited to preventive inoculations, in which office they have given brilliant results in variola and rabies. The value of Homœopathy is not deteriorated by the antiseptic.

Dr. J. P. Tessier combats these conclusions, and says that perfection in a new science, such as bacteriology, should not be expected. Its partisans do not consider that all diseases are produced by a pathogenetic microbe, but reserve this etiology for infectious diseases.

They do not advocate destroying the microbe, but the placing of the organism in a condition that will impede reproduction.

If the partisans of the microbe doctrine should choose a formula they would ultimately have to adopt the law *similia similibus curantur*, as in the case of Pasteur and Galtier's methods for treating hydrophobia and that of Chauveau and Arloing in treating septicemia, which are mere applications of that principle.

There is no doubt that microbes cannot operate without the existence of a defined predisposition, but they have a capital importance, and syphilis, variola, measles, etc., do not appear spontaneously but are the work of a live organism.

It is evident that the antiseptic is all-powerful in surgery. It cannot be predicted to what results it will some day attain. In medicine it is yet in its infancy. We cannot say it will not go any further.

Homœopathy is the greatest discovery in medicine. It has suffered nothing by this new doctrine. It not only combats virulent diseases, but every kind of morbid condition.

Dr. Dudgeon, of London, believes it is difficult to prove that the microbes are the cause of diseases, and that if they were, the antiseptic therapeutics would still be indefensible; for in killing the microbe the danger is great of killing the patient. Many of the best English surgeons have renounced Carbolic acid, as it is liable to accidents, and they believe that exquisite care and scrupulous cleanliness are sufficient. Bolle, of Aix-la-Chapelle, has replaced Lister's method by the use of cotton soaked in Alcohol and tincture of Arnica. He makes many cures and obtains good results.

We should be careful not to exaggerate the value of Pasteur's results in the treatment of rabies, for Dr. Kranzinski, of Moscow, has reported three hundred and seven cases of bites by rabid animals in which no preventive inoculations were made. Yet only 8 or 2.6 per cent. died. On the other hand, in England two hundred and fourteen have been treated at Pasteur's Institute, of which 3.27 per cent. have died. With these results what is the value of inoculation?

Dr. de Brasol, of St. Petersburg, does not consider preventive inoculations as the ideal prophylaxis. According to this idea the healthy man must be inoculated with all contagious diseases, variola, cholera, typhoid dysentery, etc. He believes that all that is necessary is to fortify and induce health in the human organism, and not weaken it and infect it by communicating the germs of all these diseases. He has arrived at the conclusion that the results of vaccination against variola are not satisfactory.

Agreeing with Dr. Jousset's opinion that the microbe theory is false, as an etiological doctrine, and that antiseptic therapeutics is impotent as a curative agent, he amplifies his views, and affirms that the microbe theory is false as a base of prophylactic therapeutics. The work of the laboratory is "*Love's labor lost.*"

Dr Villers is a microbist, as is Dr. Tenier. Both, however, attribute great importance to the homœopathic treatment even in surgical cases. For this reason he recommends Dr. Bolle's treatment, saying it does not affect the action of our medicines. It consists of cotton soaked in Alcohol with 1-100 of tincture of Arnica. The cotton prepared with this solution is put in position and moistened daily without displacing it.

Dr. Clark, of London, observed that individuals poisoned by Carbolic acid show symptoms analogous to the complications of traumatism, for which reason it can be said that the methods of Lister work according to the law of the similars. The session closed at 6½ o'clock.

### The Homœopathic Materia Medica.

*Thursday, August 22d.*—Morning Session. President Dr. P. Jousset in the chair. The minutes of the former session were approved.

Dr. P. Jousset thanks Dr. Dudgeon for his contributions to the discussion given at the last session. He maintains that antiseptic medicines are supported by the laws of the contraries when they pretend to be curative. The preventive inoculations, badly named vaccinations, are really founded upon the law of the similars although constituting the practice of isopathy. The orator defends Jenner's practice of vaccination as an almost sure preventive of variola and does not think it dangerous.

On motion of the President, it was decided that the papers sent by absent members should be considered after the regular order of the day. Dr. Gailliard read his paper on *A method for study of Pure Materia Medica.* Hahnemann's works on materia medica form three volumes of the *Materia Medica Pura*, and the *Chronic Diseases* form two volumes. Allen's *Encyclopædia* is much more considerable, and lastly, in his dissertation before the Royal Medical Academy of Belgium, in 1877, the speaker added to the provings of Arsenic and Phosphorus nearly eleven hundred observations, all of allopathic origin, that confirm the symptoms of Hahnemann's pathogeneses.

He afterward did the same work for Belladonna and Digitalis.

The observer and the experimentor in materia medica should follow three ways of study essentially distinct, and that are all complete in one: analysis, synthesis, and comparison.

The pathogenetic studies of Hahnemann and his followers are incomplete and difficult to read because they have been produced exclusively by the analytical procedure.

### A Plan to Reform the Materia Medica.

A reform is needed in the materia medica by the undertaking of works of synthesis and comparison. Once done it will be much more important and oftener consulted than purely analytical work that is useful for proofs only.

*Analytic study* investigates chemical and anatomical modifications of the organs and tissues as well as the functional modifications produced by a simple pathogenetic agent.

*Synthetic study* establishes that medicines are morbific agents, and that the nature of the cause alone distinguishes whether the condition is from natural disease or from the effects of the medicines. We, therefore, must study their invasion, their evolution, their lesions, and their complications, and thus hit upon the correct diagnosis.

*Comparative study* consists in the comparing of the character of a medicinal disease with that of a natural disease, and with other medicinal diseases, establishing thus its differential diagnosis.

These investigations should be verified first in the well man; secondly, by application upon the sick. But such investigations must not be confounded with the *ab usa in morbis.* Lastly and before all, in animals.

These complex investigations are almost beyond the power of one man. It would be most profitable to have them done in common—that is to say, by a committee of all the delegates of the different countries.

The Congress could accept this proposition and order the work begun. The methods of its execution could be studied before the future Congress of London.

Dr. de Brassol does not believe that such investigations are necessary, above all, in animals, as in such a case they could not give us the subjective symptoms which the experiments in the well man furnish us. There are also differences in the action of medicines according to the animals experimented upon. How much more then between the action on animals and the action on man.

Dr. Batault advises, above all, the study of physiological anatomy of the cells, giving an account of their vibratory method of action. Can we reach this *desideratum?* It would be most difficult to employ this method in the nervous system, as the nervous cells differ slightly except in their functions. For all nervous disturbances, and for many diseases that exhibit none or else only insignificant lesions, analytical experiments upon the well man are absolutely necessary. Studying clinically the diseases of the nervous system, it is necessary to investigate which remedies produce analogous symptoms to those that are observed in the sick, and to infer from this that a medicine has a given action or power over a determined group of nerve cells.

Dr. Cigliano believes that what is necessary for the materia medica is the method of explanation of the symptoms consisting in studying the following different circumstances: prodromos, the qualities of the symptoms, similitudes, modalities, concomitants, increasing and diminishing circumstances, conditions, and habits. He has applied this method to the work that he has published, *Individualization of Medicines for Symptoms and Diseases; or, Great Homœopathic Clinical Repertory.*

This method would give the materia medica a classification of which the pathogenesis of *Aconite* as presented to the Congress is an example.

Dr. Léon Simón approves in all its points of Dr. Gailliard's proposition. The author knew in his youth the homœopaths of the first generation, who studied the *Materia Medica* much more than we do now, and proclaimed highly the efficiency of Homœopathy; whereas, those who have come later and who have simplified the materia medica come to a contrary conclusion.

It is true that Hahnemann's *Materia Medica* is not perfect, but it is easier to criticise than to imitate. What they should do is to try to complete it. Indeed, Imbert Gourbeyre has partly done this for Arsenic and Belladonna.

Synthesis is good, and Hahnemann has made synthesis the head of each chapter devoted to the different medicines, but the patient is a being essentially analytical. Therefore, it is necessary to analyze individually, and, as Hahnemann said, cure not the disease, but the patient.

### A Plea for the Cyclopædia of Drug Pathogenesy.

Dr. Guerin Meneville, speaking for Dr. Hughes, who distrusts his own knowledge of the French language, says that he approves of the plan of Gailliard, but before commencing these studies it is necessary to collect data. This work has been rehearsed in the *Cyclopædia of Drug Pathogenesy*.

He presents the Congress with the two parts of this work that have already appeared, extending to *Natrum muriaticum*.

The conviction has been established that the homœopathic materia medica is far from what it ought to be. The compilations of Jahr and of Noack and Trinks are not clear. Dr. Allen's *Encyclopædia of Pure Materia Medica* has two capital defects that will prevent its being the materia medica of the future. First, it contains all symptoms—good, bad, and indifferent, and secondly, it persists in the pure conceptive (*esquematic*) form of explanation adopted by Hahnemann. The symptoms isolated from such concepts (*esquemas*) are completely incomprehensible.

In the *Cyclopædia*, whenever possible, the exposition of the pathogenesis is a narrative of symptoms caused by drugs, followed by clinical cases, giving thus a force and character analogous to the descriptions of idiopathic diseases. This is fixed in the memory, and is completed by means of experiments on animals.

It has been necessary to select the authors, throwing out entirely such novelists as Houat, Wolf, and Mure, and yet earnestly approving of the symptoms of Hering and Peters.

In relation to toxic symptoms, it has not given an extensive collection, but much better, only the typical symptoms of distinct forms. Using various abbreviations, easily intelligible, all the materia medica since Hahnemann could be condensed into four volumes of ordinary size. Hahnemann's pure *Materia Medica*, translated from the latest editions with its preface and annotations, would make up the first volume.

Dr. Léon Simón thinks that the work that Dr. Hughes describes seems to correspond closely to Dr. Gailliard's programme, and he favors its completion.

Dr. P. Jousset makes the observation that the opposition of views that seems to exist between Drs. Gailliard and Léon Simón is more apparent than real, as the first recommends the synthetic study of materia medica, yet wishing that analytical study be preserved entirely. When the symptoms are described in the order of their appearances we obtain a species of synthesis that gives more important instruction than the symptoms absolutely isolated by Hahnemann.

If what was said lately by Dr. Léon Simón is true, our antecedents knew more of materia medica than we do. It is probable we are not as assiduous in its study on account of its colossal proportions. Perhaps the explanation may be that formerly the homœopaths were less called upon to combat acute affections, treating in preference chronic diseases. As a practical conclusion to this discussion, we should study the materia medica with greater care.

The President decided that Dr. Gailliard's proposition had been approved by the Congress.

### Electro-Homœopathy.

Dr. Conan read a paper on the employment of electricity in homœopathic medicine, in which is used successively mineral electricity, vegetable electricity of sensitive plants, of electrified complex medicines and external medicaments.

Hahnemann studied symptoms produced by the north and south pole of the magnet. The speaker has prepared globules of a mixture of equal parts of Alcohol at ninety per cent. and of water in which he has submerged a needle magnetized by the north pole of a strong magnet or loadstone. One globule of *Mimosa pudica* alone soaked in this magnetized water cured in a few hours a lady prostrated by an invincible chronic diarrhœa.

I cured also a man forty-six years old, who was in a debilitated and melancholy state, and who showed all the symptoms of cerebral softening. He had tried all other treatments in vain. I administered water electrified with a negative electrode of gold by means of a statical apparatus. I believe the electro-homœopaths should make use of vegetables united with minerals, and electrified by means of a strong induction machine. The vegetables that should enter into their composition are those of an especial vitality, such as the sensitive plant, whose leaves close on the slightest contact—the *Sparmania Africana* whose anthers are irritable; the parietaria, the nettles, berberis, the filaments of whose stamens are agitated when they are touched by the point of a needle and fold over upon the pistil. All this can be called vegetable electricity. The electro-homœopath has artificially electrified these electrical plants.

Belotti, in his preface, indicates the complete way of preparing these complex medicines. He says that the solubility of the insoluble substances that have to be triturated is augmented by connecting the solvent with two opposite poles of an electric current.

Dr. Conan deduces that an investigation would be useful of the employment of magnetized alcoholic water or of electrified medicines, determining the character of the electric current that should be used in any given case, whether a continued or an induced current, and whether through thick or thin wire.

Dr. Léon Simón finds in this paper two subjects to consider: First, the employment of electricity—an agent whose use is extending daily more and more in medicine, but generally confined to specialists. Second, the employment of electricity in connection with Homœopathy, the discussion of which will take place to-morrow in the considering of two communications on Mattei's remedies.

### Polypharmacy, or Mixed Prescriptions.

Dr. Gailliard related the discovery of the use of complex medicines:

Ægidi, friend of Hahnemann, wanted to administer two or three medicaments together having a similar action, and made vain attempts to form a school. Later, Lutze actually practiced this method. But only after thirty years do we find that the polypharmacists have completely systematized their methods.

About the year 1850, a poor abbé of Turin, named Soleri, practiced Homœopathy, availing himself of the small manual of Jahr. One day he gave a miserable peasant several different powders to take successively for a period of forty days. His patient, desiring more speed than certainty, swallowed it all at once, and was cured before the forty days had expired. To the abbê this seemed a miracle, and forthwith he advocated the complex system, and proclaimed the superiority of it over Hahnemannism.

He formed a partnership in 1861 with his nephew, Dr. Belotti. They classified remedies in twenty-six series, the cerebral, medullary, great sympathetic, vascular, lymphatic, etc. In 1866, Dr. Finella simplified Belotti's method and created new formulas, specifics against worms, etc.

Signor Mattei, who was then an unbeliever, conceived his idea of Electro-Homœopathy. This method was revealed to him by Providence, the secret of which he guarded with great care, no doubt by Divine command. We know to-day, thanks to Sauter and other brotherly enemies, the composition of his pills for scrofulosis, aneurisms, etc., and his electric solutions, red, green, etc.

The latest incarnation of complete polypharmacy has been revealed to us in the year of grace 1888, in that "Homo-homœopathist," Dr. Conan. This "Homo-Homœopathy" comprehends twenty-six series of medicines, all specifics; anti-febriles, specifics for inflammatory diseases, acute or chronic diseases of the cerebrum, meningitis, etc., etc.

Each series of medicines contains invariably six groups and each group embraces from twelve to thirty remedies. The groups should be alternated on various occasions each day—that is to say, the series should be alternated and be employed successively in divers dilutions; and, finally, from time to time as an intercurrent, the *urinary* simillimum dynamized in a very high dilution the thirtieth, one hundredth, two hundredth, three hundredth.

The session closed at quarter after twelve.

### Progressive Locomotor Ataxia.

Afternoon Session. The President, Dr. P. Jousset, in the chair. The proceedings of last session were approved.

Dr. Villers opened his paper on *Homœopathic treatment of locomotor ataxia and pseudo-tabes.* In all his cases he has observed that the disease is to be attributed to syphilitic infection. At times a cold taken from dampness, he judges, might be the immediate cause. In such cases he finds indications for *Rhus-tox.*

The diagnosis of true ataxia may be difficult, as certain cases of hysteria present similar symptoms. From the therapeutic view, ataxia can be divided into two periods: first, the irritable, that corresponds to medullary inflammation, during which time complete cures can be obtained: second, of sclerosis and atrophy, which are incurable. *Secale cornutum* is the most important medicine since the works of Tuczek have demonstrated the simillitude between the symptoms of ergotism and of tabes. Darting pains are the symptoms most troublesome and most pointed. Dr. Villers has observed that they are aggravated by light contact and alleviated by strong pressure. *Graphites, Sulphur*, and *Stannum* correspond to these pains, which are administered from the thirtieth up to the two hundredth in single doses, waiting the full effects of the remedy before changing.

The symptom most frequent is formication that gives way to *Secale* or to *Nux-vomica.* A sensation as if drawing around the waist, which indicates *Graph., Nux-v., Stannum*, and above all *Rhus* and *Alumina.* Sensations of heat or cold at limited points in the skin that are helped always by massage and hydrotherapeutics. The conditions of sexual excitement, more active in woman than in man, might be symptoms of hysteric-pseudotabes. *Sulph.* is suitable, when these ideas pursue the patient even during work.

Impotency might be modified with advantage by *Tabacum*; above all when it comes accompanied with great weakness of the knees. Tobacco is almost as important as Secale-cornutum in the treatment of tabes, and the author saw in his service at North Angel a patient, a man who for many years had presented all the signs of locomotor ataxia, but who was really affected with nothing but nicotine poisoning.

Constipation gives way to Nux-vomica, Opium, and enemas of tepid water. Retention of urine is to be avoided, according to the author. Compression of the hypogastric region with the hand will cause contraction of the bladder and discharge, thus avoiding the use of the catheter. Among the adjuvant measures of treatment, electro-therapeutics should be discouraged, as the action of homœopathic medicine is disturbed in tabes by such treatment. But, on the contrary, baths are advantageous; however, Carbolic acid baths, lately so recommended, seem useless. Mineral waters are indifferent, though such waters as Gastein are advantageous, but great prudence is to be recommended in their use; at the most the patient should have only one or two baths weekly.

Dr. Vincent Léon Simón thinks that when darting pains are aggravated by light contact, and relieved by strong pressure, *Plumbum** might be tried, founded upon the localization of symptoms in the inferior parts of the body. *Zincum* has had desired effects in urinary disarrangements.

With relation to the neutral mineral waters, La Malou, in France, has a favorable action, which is comparable to that of Gastein.

Dr. Daniel, of Marseilles, is surprised that Dr. Villiers has not spoken of Arsenic among the medicines for tabes. It is the agent that makes the waters of La Malou most efficacious.

Dr. de Brasol advises *Agaricus-musc.*, when the darting pains are accompanied by cold sensations.

Dr. Batault has cured them with Bryonia$^{30}$.

Dr. Gallavardin, with one dose of Nux$^{30}$ has enabled patients with locomotor ataxia to walk erect, and in the dark. He has often cured urinary derangements with Conium$^{600}$.

Dr. P. Jousset said: We possess to-day sufficient data for the treatment of locomotor ataxia by a medication that may be called *Coördenada.* The first period only is susceptible of cure. For this there are two remedies above all others, *Sulphate*

---

* Pains which are aggravated by light pressure, and ameliorated by strong pressure are characteristic of Nux-vomica. We have had brilliant success in relieving a case of renal colic by attention to this symptom.—W. M. J.

*of Atropine*, recommended for some time by Dr. Hughes, and *Sulphate of Strychnine*. These remedies are given in third and second triturations, never lower. In the pathogenesis of Belladonna and Nux-vomica, we find a faithful picture of the symptoms of ataxia in its beginning. Clinics have confirmed these statements, and show that this treatment is efficacious. It is difficult at times to recognize which of the two remedies is best indicated. One could be used during fifteen days, the other the fifteen days following, and thus successively. As adjuvant treatment, sea-bathing appears to be very favorable.

### Insomnia and Neuralgia.

Dr. Mark Jousset read a paper on *Massive doses of Caffeine in the Treatment of Insomnia and Nocturnal Neuralgias*.

Insomnia, with agitation, is one of the effects of coffee and of Caffeine. Coffee sometimes also produces neuralgic symptoms in the inferior maxillary, the teeth and the stomach, producing as well hemicrania.

Here, then, is an application of the law of similars in the use of coffee and Caffeine against nightly neuralgias and insomnia. Caffeine in massive doses, five to ten centigrams of the crystals, seems necessary at times in some cases. Such doses have cured two facial neuralgias, zona of the brachial plexus, two left sciaticas, and a left intercostal neuralgia.

Dr. Tesser asks if he has had any failures.

Dr. Cigliano laments that there has not been more preciseness in the individualization of this medicine, and inquires if the neuralgic pains are aggravated by contact.

Dr. Mark Jousset has had failures when he has used Caffeine in cases of nervous insomnia without nocturnal neuralgia. Up to the present time it has worked well in the treatment of nocturnal neuralgia, insomnia, intense restlessness, and impossibility of keeping in bed.

### Iritis and Irido-Choroiditis.

Dr. Parenteau read his paper on *Iritis and Irido-Choroiditis in connection with uterine affections*. Puberty, pregnancy, the menopause, and uterine lesions might engender one of these ocular affections. Puberty produces, above all, dynamic perturbations, pregnancy and uterine affections occasion *iritis and irido-choroiditis exudations* with multiplied lesions.

The author gives his attention by preference to a variety of these affections; glaucoma (*vitreitis*) an obscure affection which is overlooked among a variety of other lesions which are more obvious, such as iritis synezisis. In girls and female children it may appear alone or accompanied by lesions so minute that glaucoma predominates. It affects most commonly only one eye, and appears at times most commonly during the menses and in pregnancy.

It is characterized by symptoms of diminution of visual acuteness that appear suddenly, and which are owing to a great number of exudations, very delicate, that float in the body of the vitreous humor. If there is not phenomenal inflammation of adjacent structures, the iris or the sclerotic, the affection is indolent. With the ophthalmoscope a fatty point more or less prominent may be seen in the fundus of the eye.

The prognosis is grave, as it may result in hopeless obscuring of the field of vision. Curing the uterine affections or re-establishing the menses might produce relief.

Homœopathic treatment produces a cure in a few weeks, even in a few days, if the patient is treated from the beginning. The principal remedy is *Mercurius corrosivus* from the 1st to the 6th. Given in small quantities it is sufficient for the cure. If at the same time there is iritis, Atropine must be injected into the eye. *Sulph.* and *Arsen.* might be indicated, but are inferior to Mercurius.

Dr. Gallavardin refers to a case where he restored sight to a lady who had a diminution of visual acuteness, and who was presbyopic, by touching the eyelid with a steel needle.

### Diphtheria and Croup.

Dr. Daniel Serrand read his paper on *Diphtheritic Croup cured by Cyanide of Mercury.*

Lily M. F., of Philadelphia, aged three years, on the 14th of August, 1880, presented a quick angina pulposa (*pharyngitis*), for which she took *Bellad.* From the 16th to the 25th she seemed to regain perfect health.

In the night of the 25th the child was attacked with croup. Both tonsils were covered by a fold of false membrane, apparently diphtheritic. The palate began to be invaded. *Bromine* interiorly by irrigations in the mouth and a spray continually surrounding the patient.

On the 26th, at six A. M., the fold that surrounded the tonsils was thick and a yellowish white, the palate was completely covered; respiration stridulous. Although weak the little one ate a little. After consultation, Dr. Cretin agreed upon Bromine every hour and Byronia every five minutes.

The 27th, the false membranes had increased. The swelling of the lymphatic ganglia was considerable, oppression very great, voice gone, and induration much marked. The dyspnœa proclaimed the approaching crisis. The question of tracheotomy was considered. Mecurius-cyanatus 2d x trituration with a spoonful of coffee each half-hour.

About the middle of the night the child was better, there was less oppression; tracheotomy was deferred. The vulva was covered with diphtheritic patches.

28th, her voice was better, the false membranes were loosened, child asks for food; the nose less obstructed, tumefaction was diminished and respiration was easier.

29th, great improvement. The false membranes were entirely dislodged. *She had two diarrhœic stools, one of which was entirely covered with false membranes.* The croupy cough had disappeared. Cyanide repeated from hour to hour.

30th, she was growing better and the 2d of Sept. the patient went to the Isle of Jersey, where she remained awaiting the time for her return to America.

Her convalescence was good. However, in America paralytic sequelæ occurred, which were cured later without leaving bad effects.

### Cyanide of Mercury in Diphtheria.

Dr. Beck, of Monthey, in Valais, is happy to be able to present to the Congress Dr. Alexander von Villers, the first patient cured with Cyanide of Mercury, who is here present. Amid the applause of his hearers, he explains how, during the despair of Dr. Villers, Senior, the dismay of Dr. Lund, the attending physician, and of the suffering endured by the child with a courage and resignation much beyond his years, the speaker suddenly remembered having read an account of poisoning by Cyanide of Mercury. Noting the similarity of symptoms between the recorded poisonings of this drug and the symptoms of diphtheria, he immediately had prepared the 6th x dilution of the drug, which accomplished the cure.

On motion of Dr. de Brasol, the Congress gave an ovation to Dr. Beck, and a vote of thanks for the great service given to humanity in this discovery.

Dr. P. Jousset asked Dr. Beck what he thought is the proper dose of Cyanide of Mercury.

Dr. Beck: I have always used the 6th since the first case in which this medicine was so successful.

Dr. de Brasol thought that the 2d trituration of Cyanide of

Mercury that Dr. Serrand employed would be a dangerous dose even for an adult.

As a physician he was an active witness of a great epidemic from 1878 to 1880 in the government of Poltawa, a terrible epidemic that devastated half of the infantine population of that region. Treating cases as an allopath, at the beginning of this epidemic, he employed astringents, antiseptics, and cauterization, etc., with disastrous results.

At that time he had professed sympathy for homœopathic medicine, which he had tried in chronic affections but never applied to such a malignant disease. Yet after so many failures he tried *Merc-cyan.*, and was amazed at the results obtained. He gave to all doses from the third trituration to the thirtieth. In some cases the third produced aggravation, and he hence prefers the thirtieth. Some cases do not yield to *Cyanide*, and then *Arsenicum-jodatum*, Phytolacca, Bromium, etc., effect the cure.

Notwithstanding all this there are some cases of septic diphtheria that resist all treatment and terminate in death. As to the doctors that make the pretence of having cured five hundred diphtheritic cases at one time, without one failure, with *Merc-cyan.* and at another with other remedies, as we cannot doubt their word, we must doubt their diagnostical capacity. These hasty affirmations considerably injure homœopathic prestige, and authorize our adversaries to doubt our cures. We must be strict with ourselves, and not forget that there are mild epidemics and serious epidemics, where numbers of sick die notwithstanding all treatment, and also that we never should present as proofs of our treatment any but serious cases.

Dr. James Love completely agrees with Dr. de Brasol's opinions. Dr. Comby, physician of a children's dispensary, declared lately at the *Société des Hôpitaux*, that he saw only ten cases of diphtheria in a year at his dispensary. Dr. Love treated only about fifteen, and his personal experience on *Cyanide*, as at most he has employed only the second x trituration, is but little satisfactory. He has cured croup with this remedy in ten cases in twelve, but he believes the successes are not owing to the mode of operation but to continued homœopathic treatment.

Dr. Serrand believes that there can be no doubt as to his diagnosis of diphtheria. The seriousness of the symptoms and subsequent paralysis prove it, and the doctors Cretin and Rémond, called in consultation, agreed in the conclusion.

Dr. Boyer has obtained notable results with Cyanide (sixth) alternated in very serious cases with bromine water 100 x according to Dr. Teste's formula, a dose of three to four drops in a little sugar and water.

Dr. P. Jousset claims that Ozanam was the first to use Bromine in diphtheria.

Dr. Cigliano has found the sixth dilution of Cyanide always sufficient for the treatment of diphtheria. In some cases he observed on the fourth day some phenomenal aggravations, such as diarrhœa, salivations, augmentation of fever, etc. He then suspended the treatment, and the patient was cured. He has not been so fortunate in the treatment of croup.

Dr. Mark Jousset made a note regarding Cyanide of Mercury that the third x is the dose most used in France, although Dr. Petit, of Rennes, has observed aggravations, and advises the use of the sixth x.

Dr. Serrand's observation is a valuable demonstration that Cyanide was effective until the laryngitis period of diphtheria. Moreover the pathogenesis of *Mercurius* contains some laryngitis symptoms.

In general phthisis Cyanide of Mercury is most convenient in the pharygitis period, and when the croupy symptoms appear other remedies must be used, sometimes alone and sometimes alternated. Bromine is really efficacious, but the patients do not like it. Dr. M. Jousset replaces it with *Spongia tosta*[1], which contains Bromine and has been recommended by Hahnemann. This medicine alone or alternated with Cyanide of Mercury has cured difficult cases of croup without recourse to tracheotomy.

Dr. Bonino, of Turin, never has cured diphtheritic laryngitis with Cyanide alone, always alternating it with Bromine. He observes that Dr. Serrand's case is of little consequence, as he did not employ Cyanide alone, perhaps because with Bryonia the diphtheria could not have extended to larynx.

Dr. Beck considers croup and diphtheria two distinct diseases. In croup death is caused by asphyxia, as it is impossible for air to reach the lungs. In diphtheria asphyxia supervenes because the blood corpuscles are altered. Croup can be conquered with *Acon.*, *Spongia*, or *Hepar*.

Dr. Sanllehy, of Barcelona, shares Dr. Beck's opinion, and says that croup should not be confounded with diphtheria, whose symptoms and duration are very different. Croup is a disease more localized—more pertaining to children, and always produced by catarrhal causes. Diphtheria depends on a general disposition that determines the formation of false membranes, and affects most the lymphatic temperament. Necessarily the treatment differs in the one case very much from the other. Croup is a catarrh ascending rather from the bronchial tubes or lungs than from the pharynx. Aconite, Ipecacuanha, Bryonia, and Mercury or some of their preparations, when there is ulceration, must be the treatment. Diphtheria, whose origin is poverty of the constituents of the blood, descends from the pharynx to the respiratory organs, and the most direct treatment he has found for the same is Mercury, and above all Arsenic.

Dr. Schoedler, of Berne, recommends, with Dr. Mark Jousset, the alternation of Cyanide of Mercury and Spongia. In the croup treatment it appears to him that the high dilutions work better than the low, and in cases where the latter have failed.

Dr. Vincent Léon Simón will not admit that croup is a local disease. It is a general disease, very dangerous and contagious. He sees no difference between diphtheria and croup except in situation. If the child's larynx is of preference affected it is a question of conformation, and of greater susceptibility of this organ in infancy.

It seems to prove the identity of both affections by the incident that diphtheria can be contracted side by side with one sick with croup, or *vice versa.*

### PARALYSIS AGITANS.

*Session of Friday, August 23d.*—The President, Dr. P. Jousset, in the chair. The proceedings of the previous session were approved.

Dr. Imbert de la Touche read a paper on *Seven Observations on Cures of Senile Trembling and Paralysis Agitans,* following with an attack upon the regimen used in neurasthenia. The most famous classical authors consider this disease incurable.

He first referred to the case of a nun cured of trembling of the hands which had lasted fourteen years. Secondly, a lady of sixty years who had during three years a trembling in the limbs that Dr. Heichelheim cured with Bell.$^{30}$ and Sulph.$^{6}$, alternated. Thirdly, a gentleman for four years had a trembling in the hands that impeded his writing or carrying a spoon directly to his mouth. *Rhus-tox.*$^{18}$ and afterward Silic.$^{30}$ helped him. He was completely cured by a dose of *Bell.*$^{18}$, and after an interval of eight days a dose of *Bell.*$^{30}$. Fourthly, he referred to an old man affected with senile tremblings, rapidly cured by Dr. Hughes with *Agaricus muscarius.* Fifthly, an old woman of sixty-one years with trembling of the head and left leg and tongue. At night she was obliged to walk to calm her agitation, which deprived her of sleep. *Bell., Nux-v., Iodium, Secale,*

and *Crotalus* failed. Then Dr. Cramoisy had recourse to Tarentula[12]; that repeated with persistency and in different alternations produced a cure. Sixth, he referred to an octogenarian, treated by Dr. Gallavardin for paralysis agitans of the leg and right arm that he cured by advising abstinence from wine and meat, and giving *Nux-v.*[30] once a week, and later every fifteen days. Seventh, a clerk who had tremblings of the head, cured by abstinence from meat, wine, coffee, and tobacco, and giving Carbo-veg.[2c].

Dr. Imbert de la Touche strongly insists with Dr. Gallavardin on abstinence from wine, coffee, and tobacco; wishing also to add meat, especially that of the ox, as, according to Leven, it excites the cerebrum and the nervous centres. These abstinences are recommended also in the treatment of neuroses, and particularly in neurasthenia.

Dr. Villers has cured a stubborn case of paralysis agitans with *Staphys.*[30].

Dr. de Brasol adds to the medicine named by Dr. Imbert de la Touche, *Mercurius* and *Plumbum.*

Dr. P. Jousset explained that according to the order of the day, the papers of Drs. Gailliard, Pellicer, and Garcia Lopez should be considered, and, to aid the discussion, Dr. Rappaz's project should be included. We should not forget that honor has always constituted the strength of Homœopathy, which is accustomed to march boldly without concealing any of its proceedings, and he begged the speakers to be tolerant in the following discussions.

### Polypharmacy and the Single Remedy.

Dr. Gailliard read his paper on *Polypharmacy.* Hahnemann came and destroyed polypharmacy that before his time ruled absolutely. According to his conceptions, mono-pharmacy consisted in prescribing one remedy only, or in the successive administration of simple remedies according to indications. Finally, never oppose but one single medicinal disease to a natural disease. The successive administration of single remedies has nothing to do with alternations or complexity of prescriptions.

The adversaries of the single-remedy principle, those who practice alternations and complex prescriptions, advocate the alternation method of various medicines—that is to say, oppose several medicaments to the natural disease. Some employ the alternation of the remedies as a resource that must disappear when the materia medica is better known; others accept it as an easy proceeding that dispenses with troublesome study and reflection ; some others look on it as a reform that must be introduced, as a principle that must be propagated. These last are the dangerous ones.

Some alternate two or three remedies, others five to ten, some alternate daily, others each hour or every two hours, invoking for the support of their doctrine some passages of Hahnemann. They pretend that the alternation of several medicines works better on the several symptoms presented by the patient than when employing one remedy alone, which will cover only a few of the symptoms, and add that when it is a case that seems to correspond to several remedies, it is hard to make the exact selection.

The remedy that seems the most homœopathic must be selected. The partisans of alternation must prove to us the superiority of their method; not by argument, but by observation. They say that if the cure is obtained by one alone of the medicines, it is a sign that the rest did not impede its operations. What do they know? How do they know that the cure might not have been probably more rapidly made with one remedy alone properly selected?

They believe that in certain alternations the medicaments mutually help, for having given under the same circumstances the single remedy and not having noted good results they have resorted to alternations. If they had selected one remedy alone truly homœopathic to the case in question, it is most probable that a good result would have been obtained.

They finally affirm that alternated medicines acquire especial virtues with new pathogenetic properties. This is precisely the best reason, adds Dr. Gailliard, against its practice. If the alternated medicines have not the pathogenetic properties of each of the separate medicines, its pure action remains obscure, and its use is in consequence prohibited.

Dr. Gailliard concludes by making it evident that the alternationist gives no rules for determinating the number of medicines that must be alternated, nor for establishing the frequency of the repetition, nor the intervals of rest, nor the duration of the alternation.

This subject has scarcely been skimmed, and we will return to it again. We have wished only to-day to raise the cry for vigilance. Defend Carthage!

Dr. Vincent Lêon Simón says the question of alternation is a question of acts only which we cannot *à priori* either accept or reject.

At the London Congress of 1881, Messrs. Martin and Bernard cited an observation in which *Nux-vom.* and *Opium* when prescribed separately had failed, and when alternated resulted in a speedy cure. The only method of resolving this subject would be to prove alternated medicines on the healthy man: until then, the question will remain in suspense.

### Mixed Medicines in One Prescription Absurd.

The same arguments could be applied to mixed medicines which Hahnemann did not absolutely condemn on condition that they were proved in a mixed state on the healthy man. Studies relating to some medicinal mineral-waters have already been made which are truly complex medicines.

Dr. Bonino says complex Homœopathy, Electro-Homœopathy, etc., made their dêbut in 1862, when Dr. Belotti decided on introducing complex medicines and lent himself to a speculation used by his uncle, the Abbé Soleri. He adds that Dr. Conan, in saying that electricity had a part in Dr. Belotti's specifics, intended to depreciate them. It is not so.

Dr. Conan observes that he has quoted passages of Dr. Belotti with evident bibliographical preciseness.

Dr. Bonino, with the object of demonstrating that remedies that enter into complex compositions do not preserve their especial properties, selects two samples, sea-water and Lycopodium. If the principal elements of the first—iodine, bromine, and chloride of sodium—are as effective in the mixture as when separated, we have then in sea-water an excellent remedy for syphilis, croup, etc., which does not prove true. And Lycopodium, containing *Silicea, Alumina, Ferrum*, etc., should have the action of Silicea on suppurations; of Alumina on the intestines; of Ferrum upon chlorosis, etc., which is not so. Complex medicines will only be admitted when their pathogenesis is made.

Dr. Léon Simón observes that there are three points to be examined: 1st. The alternation of medicines; 2d. The mixture of various homœopathic medicines in one prescription; 3d. The mixtures sold as secret remedies. He wishes, before all, to record

### The Three Essential Precepts of Homœopathic Therapeutics.

First. That a medicine is not truly appropriate for a determined case except when it can produce in the healthy man the same symptoms as the diseased one presents. Second. That the substance should be administered in the smallest dose possible. Third. That it is important not to have recourse to a second therapeutic agent until the first has exhausted its action. We cannot thus absolutely condemn the alternation of medicines in acute diseases; their rapid progress and sudden and repeated transformations, in which the medicinal action is rapidly spent excuses the giving of two medicines, one after the other, at proximate intervals.* A different thing happens in chronic diseases. The transformations are slow and the effects of therapeutic agents are prolonged during many days. Mixture of medicines in the same dose is truly more contrary to homœopathic precepts. All homœopaths of the first generation and those who are vain-glorious in being their disciples have never departed from monopharmacy.

### Denunciation of Mixed Prescriptions, etc.

In such mixtures the remedy never acts as it would if alone. Each medicine is a force and the reunion of all forces constitutes a result. If its physiological effects are not known, if this mixture in question has not been proved on a healthy person, it is impossible to apply it according to the laws of the similiars, and this makes it no longer a homœopathic medicine.

Before the compound formulas can be accepted it should be demonstrated in virtue of what principle the mixtures have been studied. And we should be shown how by the action of mixing several medicines their antidotal relations are avoided and even their chemical reaction. Until then to use complex medicines would be walking in the dark.

As to Electro-Homœopathy, it must not be confounded with Dr. Conan's experiments. What shall we say of them now when we treat of *secret remedies?* We know nothing of their composition nor their methods of preparation. They talk of white, red, and yellow electricity, etc., but most certainly no one has ever bottled electricity. Such remedies are not electricity.

Let them take whatever name they please for the system they recognize, but not that of the method which Hahnemann taught.

---

*The giving of medicines in sequence—that is, one after the other as indications arise, even though at short intervals—is not the same as alternation, and cannot be used as an excuse for practicing it. Just *how* short these intervals between repetitions must be will depend upon the closeness of the adaptation of the remedy to the condition of the patient, the confidence of the doctor in his own knowledge, and his courage in awaiting its effects even through an aggravation. Thus the knowledge and personal qualities of the doctor are drawn upon, and it is the value of these qualities that stamps the man and raises him to a high rank among his fellows in the profession, *determines his professional standing.* This was the secret of the success of the late lamented Dr. Adolph Lippe.—W. M. J.

All of us that accept the title of homœopathists and bear it with honor, declare "that homœopathic therapeutics does not contain any medicine whose physiological effects have not been verified by proving upon the healthy man, and whose pathogenesis is not well known." We loathe and condemn the fabrication, sale, and use of medicines whose nature, composition, and method of preparation are secret. Call themselves what they will, those who recommend such practice, (say) Electricians, if it please them; but let them leave us our title of homœopathists and Hahnemann's work the title of Homœopathy.

### A Plea for Alternation.

Dr. Dudgeon says that monopharmacy is the theoretic perfection of homœopathic practice; but it is not always practicable, or cannot always be practiced; as diseases, being almost always complex, we need to use several remedies at a time, and it is not just to say that Hahnemann was an enemy to polypharmacy. Ægidi having proposed on certain occasions the mixture of various remedies, communicated his ideas to Hahnemann, who embraced them with distinction. Dr. Dudgeon has just translated some of Hahnemann's letters, written between 1813 and 1843. In one of them he speaks approvingly of the proceedings of Ægidi, and says that *Hepar-sulph.* is a composition of two medicines. Dr. Dudgeon believes that Hahnemann was not really so dogmatic as some of his disciples who are called Hahnemannians. Dr. Gailliard has made good arguments in favor of the administration of one medicine, but there are also equally good reasons in favor of the contrary.

Dr. Sanllehy thinks there is an easy solution of the case. Alternation must be distinguished from complex prescriptions. Hahnemann never opposed alternations, as he advised *Rhus* and *Bryonia* in typhoid fever. There is not perhaps one homœopath who does not alternate medicines. Hahnemann advises, in the first and second editions of his *Organon* the single remedy. In the third it was not such an affirmative, owing perhaps to Ægidi's influence; but he again did so in the fourth and fifth editions. The complex Homœopathy of Mattei, Sauter, etc., is mere quackery, and we might as well collect in one mixture alone all known medicines to oppose all known diseases, as to make a number of groups of remedies, as they do to-day.

### Dr. Conan's Defense of Mixed Prescriptions.

Dr. Conan says: He alone is defending complex Homœopathy attacked by all the preceding speakers. He has been induced to leave the method of the single remedy to employ complex medicines, by the method of Dr. Brunner's book, called *Medicine Based Upon the Examination of the Urine.* Seeking to discover a means to determine the selection of medicines, he examined the urine with a microscope, and was able to destroy the abnormal organic elements it contained with vegetable or mineral tinctures, adding a drop of sulphuric acid. He noted that ordinarily this examination of urine indicated three or four medicines, and he was thus induced to prescribe the remedies together.

Dr. Conan's investigations have demonstrated to him that various remedies are always necessary to cover a morbid state, and he has recognized that this grouping obeys a particular law —relative antagonism. Classical homœopathic books repeatedly recommend the administration of such a medicine after such another, and frequently the second remedy is the antidote of the first.

Dr. Conan deduces from this that complex medicines are demonstrated by clinics, by materia medica, and the human organism, and that a group of medicines is generally composed of substances that have in themselves an action analogous and antidotal.

Dr. Chapiel, of Bordeaux, asks if Dr. Conan's mixtures have been experimented with on the healthy man.

Dr. Conan replies that, knowing individually the action of each element of the mixture, he can deduce the action of the mixture in conjunction.

Dr. Chapiel says the employment of substances not proved in the healthy man is quackery, not Homœopathy.

Dr. P. Jousset notifies Dr. Conan that he has already spoken more than twelve minutes, and also read a pamphlet; he begs him to finish.

Dr. Conan says that his contradictors have enjoyed a greater latitude of time, and it is impossible in a few minutes to answer objections, expound his thesis, and finish.

### A Declaration for Pure Homœopathy.

Dr. Cigliano begins by the remembrance of Hahnemann's hatred of polypharmacy, secret or divulged, whose promoters ignore the true concepts of Homœopathy. Hahnemann considered that pathology and physiology should remain subordinate to true medicine, which consists in experimental study of the remedy, and the selection of the same by the bedside of the patient.

For the selection of the remedy we must know the individuality of the symptoms of the patient, or what Hahnemann called the totality of the symptoms. This study of the symptoms must not be confounded with the pathological diagnosis, that gives a forced, stereotyped picture of disease. The most important part of medicine is the experimentation with medicines on the healthy man. This materia medica has for type pharmacological disease, and for law the individualization of symptoms determined by differences, conditions, and equivalent particulars for each medicine. Therapeutics have for their principle the law of the similars, and finally the selection of *one* medicine. Compound medicines are not worthy of consideration, as they have never been clinically proved nor experimented with upon the well man.

Dr. Cigliano hopes that this assembly of representatives of all nations will hoist very high the homœopathic banner, the only one in medicine that attacks impostures, unmasks charlatanism, and, finally, the only one that conduces to medical revolutions.

### Repudiation of Electro-Homœopathy.

Dr. Jousset proposes to the Congress that they as a practical proposition vote the following:

*The members of the International Homœopathic Congress consider that Electro-Homœopathy consists in the administration of complex medicines, that in this form have not been experimented with upon the well man, and whose compositions and method of preparation are not exactly known. They condemn this doctrine, and declare that they have no relation whatever with Homœopathy.*

The vote was carried unanimously.

Dr. Wright, delegate of the American Institute of Homœopathy, announces that, together with himself, Professors Tod Helmuth, of New York, and McClelland, of Pittsburgh, have been chosen delegates. He does not consider himself a stranger in Paris, as in 1856 he studied in the clinics of Tessier, of Teste, and of Dr. Jousset. Europe gave the knowledge of Homœopathy to America, and there it has flourished and extended rapidly. The American Institute of Homœopathy sends a cordial invitation for the International Congress of 1891.

Dr. Jousset submits the following propositions, that were adopted:

The committee on organization are recommended to publish the proceedings and the papers according to the disposable funds, publishing all or those that have been subjects of discussion.

The committee is recommended to nominate a committee on pharmacology, according to M. Ecalle's proposition, as shortness of time has not allowed its discussion. Also nominating a commission charged with preparing for the Congress the question of the laws of doses, and the method of studying the materia medica.

After Dr. Jousset's thanking the assembly, the session closed at a quarter to seven.

